# Supplementary material for: The earliest evidence of high-elevation ice age occupation in Australia
Source: Nat Hum Behav. 2025 Jun 16;9(12):2471–9. doi: 10.1038/s41562-025-02180-y (PMC12727511; doi:10.1038/s41562-025-02180-y)
Supplement: Supplementary file 1 — Supplementary Text—pollen records, pXRF results, use-wear results, rock art analysis, Figs. 1–14, Tables 1–7, code, age models and References. [file 41562_2025_2180_MOESM1_ESM.pdf]

nature human behaviour

Supplementary information
https://doi.org/10.1038/s41562-025-02180-y

The earliest evidence of high-elevation ice age occupation in Australia

In the format provided by the authors and unedited

Supplementary Text – Pollen records

Four sediment samples from square 10E were processed for pollen analysis, two from Layers 12 (150 cm) & 11 (110 cm), representing the Late Pleistocene, one from Layer 8 (80 cm) at the Early Holocene and one from Layer 5 (50 cm) at the transition to the Late Holocene. Preserved pollen concentrations were extremely low (Supplementary Table 4). The only pollen type with counts greater than 1 in the Pleistocene sediments was Poaceae. At 80 cm depth, the presence of trees and shrubs may be indicated by the presence of Myrtaceae, Ericaceae and *Casuarina* pollen types. Given the low pollen abundances, we cautiously interpret our data as indicating a transition from an open herb/grassland environment to woodland/forest from the Late Pleistocene to early Holocene.

Elsewhere in the Blue Mountains, evidence from a pollen record at Gooches Crater, around 5 km north of Dargan, indicated the dominance of Myrtaceae pollen by 14,000 yr BP[25], however a recent reappraisal of dating of this archive[17] moves the date of these sample depths closer to 6,000 cal. BP. Based on this reassessment, we argue there is no contradiction between our data and these previously published records. Similar chronological issues may exist with a pollen record from Mountain Lagoon[18], located around 30 km east of Dargan and situated at around 500 m above sea level (ASL). At this site, the authors argue that there is no significant variation indicated in the pollen spectrum from 20,000 cal. BP to present, with Myrtaceae being the dominant pollen type through the record. Given the relatively short depth of the core (1 m) and dates acquired on bulk-sediment samples around 10 cm thick it is likely that the modelled ages of this record are over-estimated[17]. At Katoomba Swamp, Newnes Swamp, and King's Tableland Swamp[19], Myrtaceae pollen are reported in low abundances prior to 10,000 cal. BP, however this is also based on dates acquired on bulk sediment and must be approached cautiously.

At Blue Lake (1930 m ASL) in the Australian Alps[20], pollen records dated to the "Pleistocene termination" (given as 18,000 to 15,000 cal. BP) are dominated by local herbaceous and long-distance pollen types, that the authors interpret as indicating a sparsely vegetated landscape composed of grass or heathland plant communities. An increase in arboreal pollen between ca. 15,000 to 11,700 cal. BP is interpreted as evidence of the shifting of the tree line to higher altitudes at the start of the Holocene. This appears consistent with other data from two sites at around 1000 m and 800 m ASL in the Southern Tablelands of NSW[21] that indicate swamp- and grassland landscapes during the LGM, with forest elements only significantly represented after ca. 10,000 cal. BP. While at lower latitudes than our site, these records support our interpretation of open, drier landscapes at high altitudes in Late Pleistocene eastern Australia.

Supplementary Text – pXRF results

The raw material composition of the complete assemblage, including quartz is shown in Supplementary Fig. 9 and Supplementary Table 5 and examples of the main raw material types in Supplementary Fig. 8. Quartz is found locally both as cobbles and bedded veins. Within the pXRF analytical sample, 66 came from the Holocene, and 43 from the Late Pleistocene strata. Combined visual and multivariate geochemical analysis revealed five different raw material

groups: claystone, an unidentified siliceous material, black quartzite, ?quartzite, and hornfels (see Supplementary Table 6). All the material groups are present in both the Holocene and Pleistocene, except for black quartzite, which was only present in the Pleistocene.

Claystone is by far the most abundant raw material in the assemblage (n = 90). It is typically fine grained, conchoidally fracturing and buff-grey to white. It sometimes displays sedimentary banding. Rarely, it is greenish in colour. This material is known from other Blue Mountains sites, where it has been variously termed "chert"[28]"silicified tuff"[47], and "Fine Grained Siliceous"[48].It has previously been attributed to the Burragorang Claystone Member (BCM)[48,49].This raw material type occurs throughout the Blue Mountains and has been recognized as far south as Bulli, in the Illawarra[50].

Other materials are far less common. The unidentified siliceous material is chemically distinct from the local claystones. It makes up a small proportion of the lithic assemblage (n = 10), with five lithics coming from the Late Pleistocene layers and five from the Holocene (see Supplementary Fig. 10). Although its closest chemical match is the Jenolan chert, which is located approximately 50 km to the south-west of the site, four of the samples were noted as having a coarse non-cryptocrystalline appearance and were identified as quartzite in hand specimen, while six were identified as chert (see Supplementary Fig. 8 B,C,F and I). There are references to "medium to fine-grained white-buff, well-bedded quartzites"[51] around the Jenolan caves, but the chemistry of those quartzites has not yet been described. As such, the chemical resemblance between the quartzite and the Jenolan cherts might be co-incidental and the provenance of this material is being treated as unknown, but exotic.

Black quartzite was the only raw material unique to the Late Pleistocene, where it was used to make two flakes and a hammerstone (n = 3) (See Supplementary Fig. 8 G and J). The black quartzite is chemically and visually distinct from the other ?Quartzite materials (described below) in the assemblage, being red brown to black, coarse grained in hand sample, and highly enriched in iron. Geochemical matches to this group have been made with river pebbles in the Hunter valley, approximately 150km to the north of the site[52]. As such, this material is being treated as exotic.

The final raw material has been tentatively classified as ?quartzite (n = 4) and is present during the site's Holocene and Pleistocene (See Supplementary Fig. 8 A). Three of these have pebble cortex. The raw material identification for these four artefacts is preliminary only, as some chemical overlap is noted between the ?quartzites and two artefacts visually identified as hornfels (#585 a large Holocene era nut-cracking pebble anvil, see Supplementary Fig. 13, and #254 a single Pleistocene flake with primary pebble cortex, see Supplementary Fig. 8 E-11) suggesting that these may represent the same material category. Both are exotic, but scarce quartzite dropstones can occur within the other Blue Mountains formations, and both quartzite and hornfels are available in the gravels of the Cox's River and other water courses nearby[53].Moreover, hornfels is common in the neighbouring Megalong valley and has been known to travel east across the mountains to the Sydney basin during the terminal Pleistocene[17].

Supplementary Text – Use-wear results

The sandstone slab (length 190 mm, width-170-185 mm, thickness 40-60 mm) showed macroscopic wear traces from use in the form of two linear grooves on one face (Supplementary Fig. 12a). The first groove (Supplementary Fig. 12a, point 1) was 100 mm in length with width from 10 to 15 mm and depth 5 mm. The second grove (Supplementary Fig. 12a, point 2) was 60 mm in length, 10 mm width and 5 mm depth. Both grooves had V-shaped cross-section.

The surface within both grooves was characterized by distinctive abrasive wear formed by the back-and-forth movement of an object resulting in slight smoothing and levelling of the wall of the grooves. Dense shallow parallel striations were oriented along the long axis of the grooves reflecting the direction of the actions (Supplementary Fig. 12b-d). Cleaning of the surface under running water removed weakly cemented rock grains with visible wear traces from the weathered outer layer of sandstone slab. However, the underlying less weathered and harder sandstone surface (Supplementary Fig. 12a, points 3-4) still displayed abrasive wear in the form of smooth levelling of the used area with rounded rock grains and shallow parallel striations (Supplementary Fig. 12e-g).

Observed use-wear patterns within grooves and the size of grooves are consistent with shaping by abrasion bone or wooden artefacts such as needles, awls, bone points, nose-bones[54,55]. Experiments with files and abraders show that poorly cemented, porous and coarse-grained sandstones are very effective at filing bone and wood[56].According to historical and ethnographic records, bone and timber were used by Aboriginal people in various parts of Australia to make points for animal skin preparation and other implements e.g.[55–57] with the hardness and resilience of bone making it a particularly useful raw material.

The basalt anvil (Supplementary Fig. 13) was located 1.5 cm higher and 35 cm west of S-ANU72609 which dated to 8,607-8,986 cal. BP. It consisted of a split river pebble with a distinctive sub-circular patch with localised dense impact marks resulting from repetitive percussion contact between the pebble’s surface, processed substance (e.g. hard woody nut and seed shell) and a hammerstone. The removal of very small rock grains in the contact area due to percussive fractures caused visible surface lowering of the used area in relation to an unused surface, or incipient pit (Supplementary Fig. 13a). The internal surface of the incipient pits was smoothed and levelled. Individual grains of the lithic raw material within the worn areas had been flattened and their edges rounded (Supplementary Fig. 13b). The used surface was also characterised by light reflective sheen and by the presence of a few fine, isolated striations (Supplementary Fig. 13b). Comparable microscopic wear traces on experimental implements made of durable stone (e.g. basalt, quartzite, well-cemented sandstone) consist of wear from cracking seeds and nuts[54,55].

Supplementary Text – Rock art analysis

Rock art was recorded via visual inspection and D-stretch photography. There are three definitive art panels located at the rear of the shelter. Panel A includes eight motifs and is located

in the north-east section of the shelter. Panel B is in the middle of the back wall and has two red ochre hand stencils with a forearm on each. Panel C has one red ochre left-handed stencil. There are some charcoal drawings that are graffiti and historical. Stencils, paintings and charcoal drawings are the major type of piment art medium in the Upper Blue Mountains[58].

The art is in poor condition, with only one stencil in Panel B in good condition. The rest are faded and generally deteriorating through micro-exfoliation and /or thin layer spalling. There is a fine dust covering on all panels, due to movement of visitors in the shelter and the continuous erosion of the shelter walls and domed roof from micro exfoliation. Insufficient charcoal is present for dating the rock art or assigning a relation with the human presence at this site. It is very unlikely to be related to the first phase of occupation.

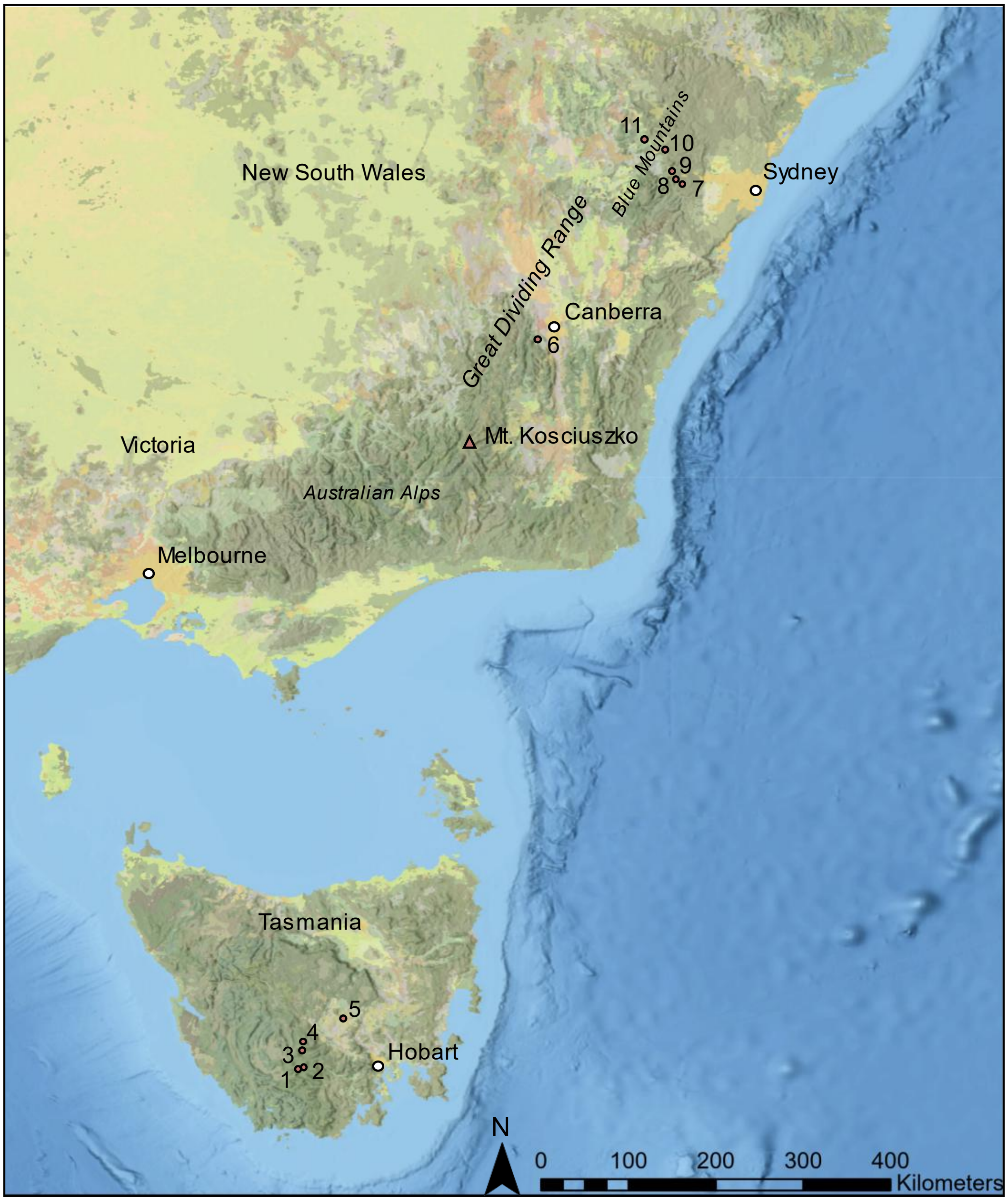

Supplementary Fig. 1

Location of Late Pleistocene sites mentioned in the text in relation to Australia's Eastern Highlands (the Great Dividing Range). The Eastern Highlands are made up of a series of mountains including the Blue Mountains and Australian Alps which run parallel to the east coast of Australia. The range begins in the north on Cape York Peninsula, Queensland and terminates in the Grampians, Victoria, with a southern spur forming the central uplands of Tasmania[1]. Sites are numbered: 1. Bone Cave, 2. Stone Cave, 3. Nunamira Cave, 4. Tiata Mara Kominya, 5. ORS 7, 6. Birrigai, 7. Kings Table, 8. Lyrebird Dell, 9. Wall's Cave, 10. Dargan Shelter, 11. Lidsdale. Site ages, elevations and latitudes can be seen in Supplementary Table 1. Base map created with ArcGIS software by Esri.

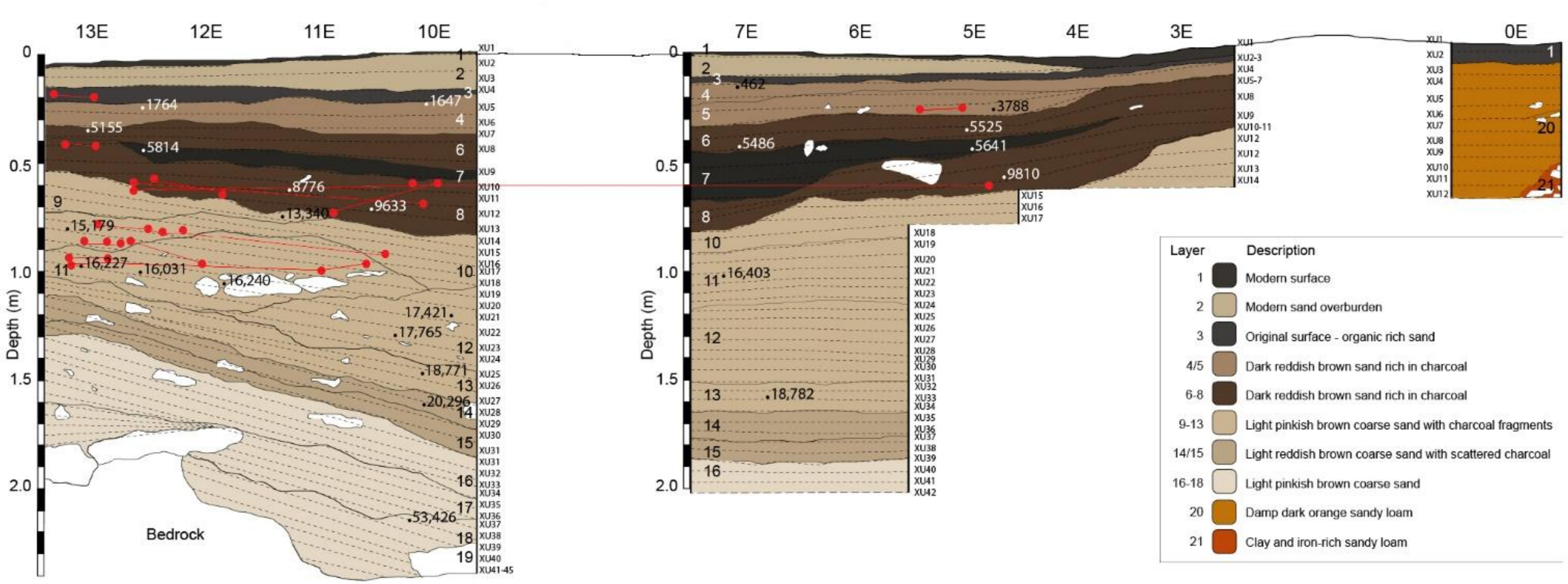

Supplementary Fig. 2

Section drawing of the southern profile wall of Trenches 1 and 2, showing major stratigraphic divisions, Excavation Units (XUs), dates (see also Supplementary Tables 2 and 3) and sediment descriptions (see also Supplementary Figs. 4 and 7). Refitted artefacts are shown as red circles connected with red lines.

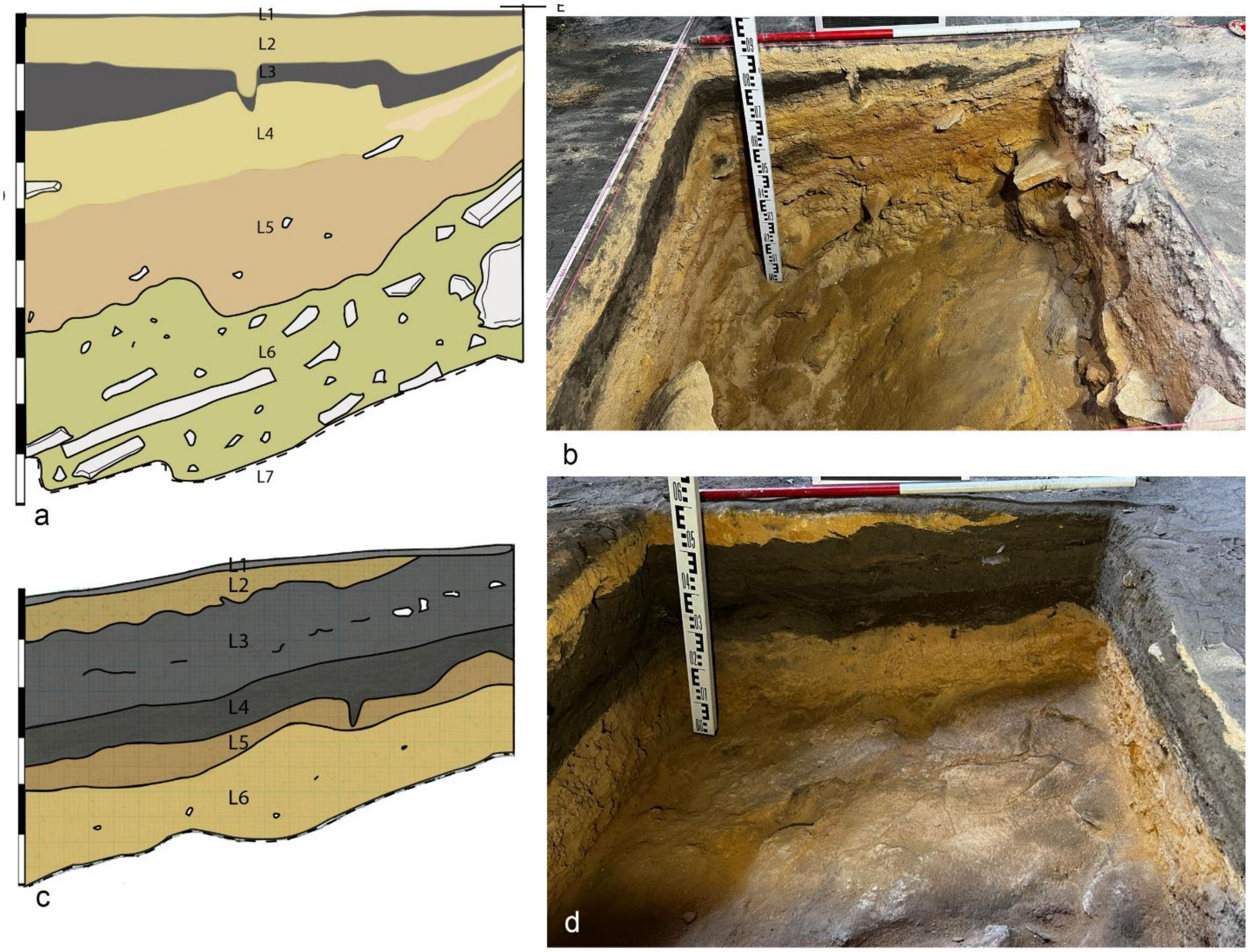

Supplementary Fig. 3

Profile drawings and photos of northern walls of Trenches 3 and 4 showing overlying redeposited sand (L2), original surface (L3), sloping stratigraphy and bedrock (L7).

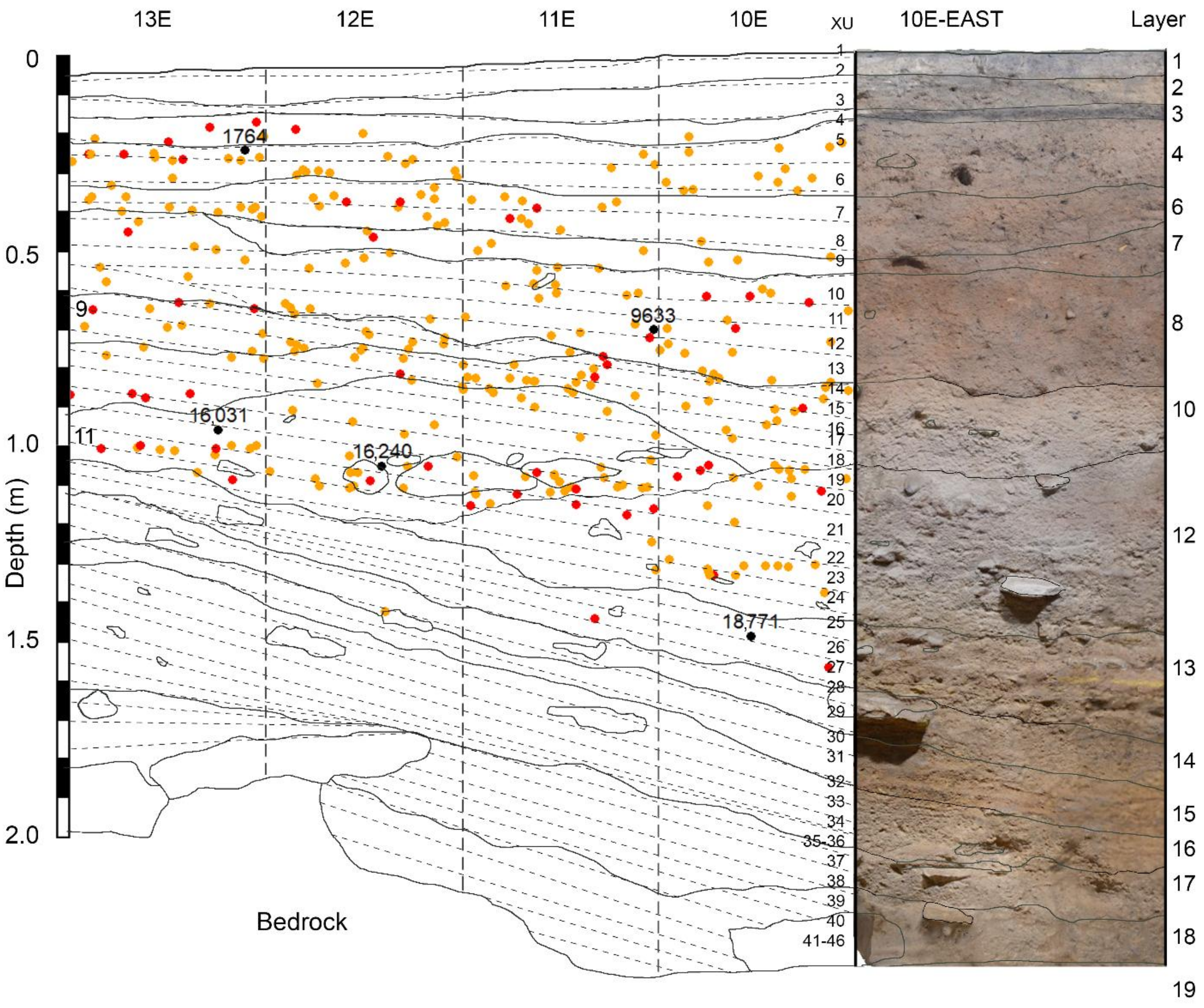

Supplementary Fig. 4

Southern profile plot of artefacts and dates found in Trench 2 in squares 10-13 from the E and F rows showing major stratigraphic divisions extending into the Eastern profile and Excavation Units (XUs). Artefacts are superimposed on the southern profile (see also Supplementary Figure 2). Plotted artefacts found during excavation are shown in red and XU provenanced artefacts found in the 3 mm sieve shown in yellow. Dates (black text) are reported as median cal. BP (see Supplementary Table 3).

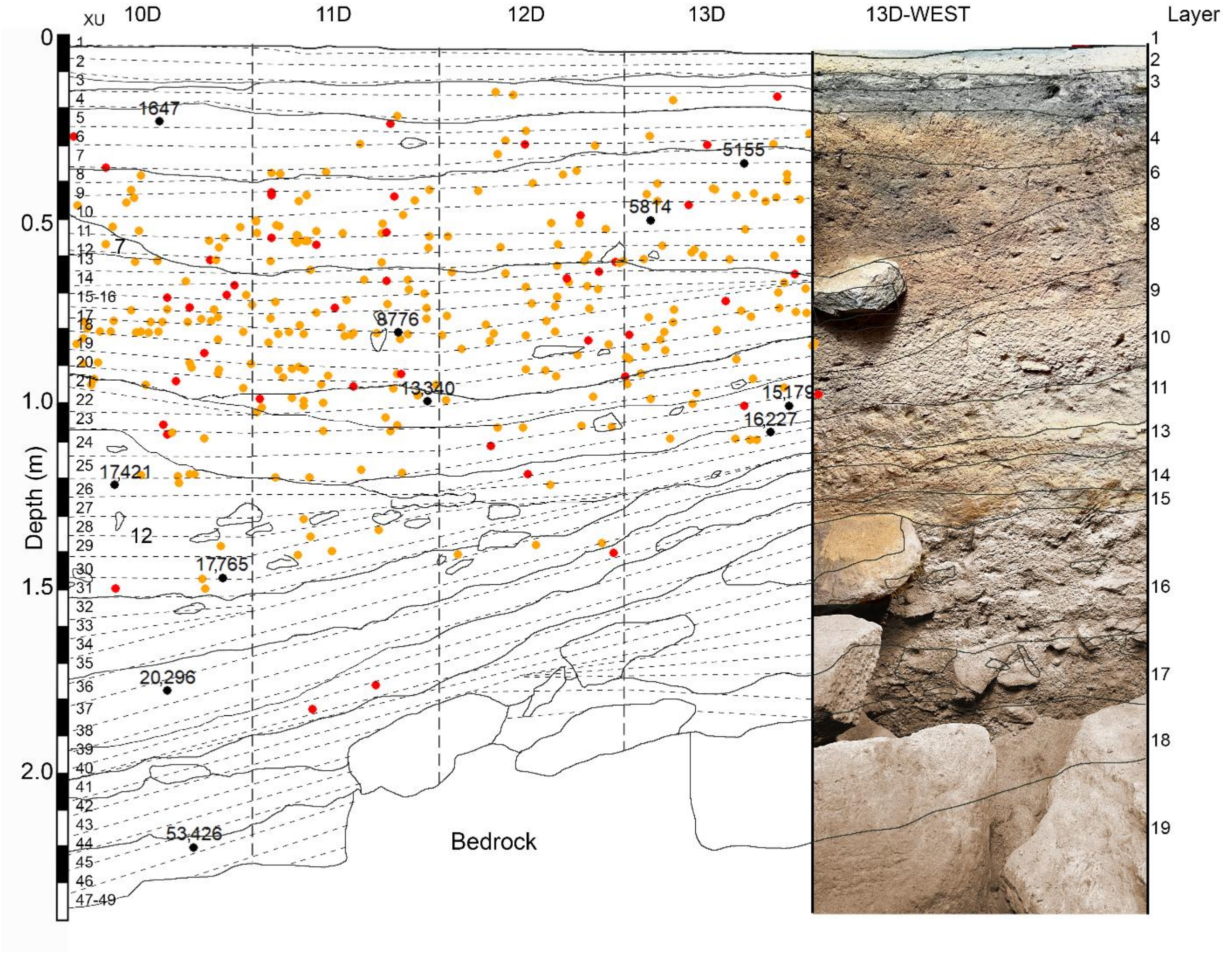

Supplementary Fig. 5

Northern profile plot of artefacts and dates found in Trench 2 in squares 10-13 from the C and D rows showing major stratigraphic divisions extending into the Western profile and Excavation Units (XUs). Artefacts are superimposed on the northern profile wall. Plotted artefacts found during excavation are shown in red and XU provenanced artefacts found in the 3 mm sieve shown in yellow. Dates (black text) are reported as median cal. BP (see Supplementary Table 3).

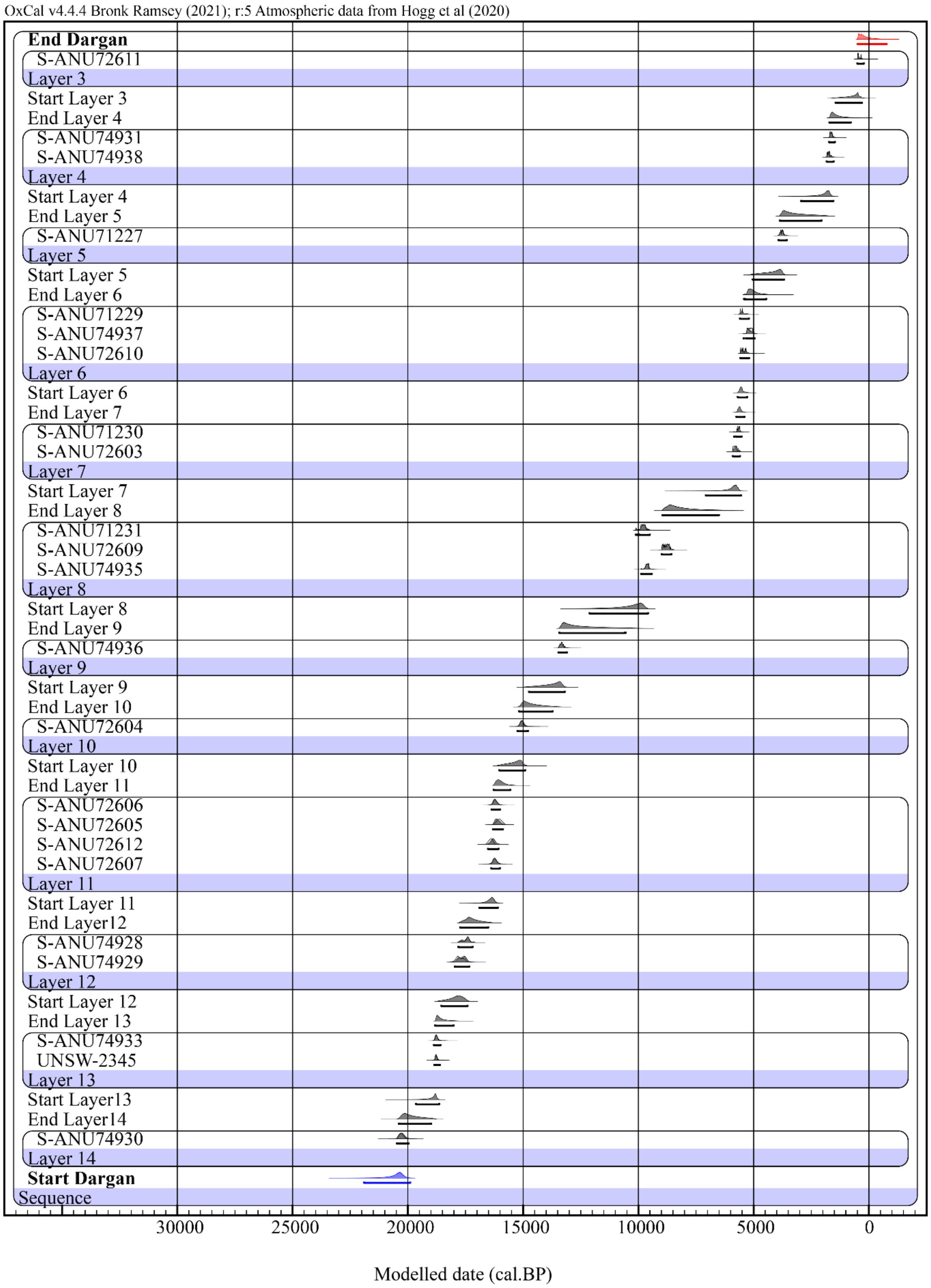

Supplementary Fig. 6

Charcoal Plus model (Model B) with contexts organized in stratigraphic order with the oldest at the bottom. Figure created in OxCal v.4.4.4.

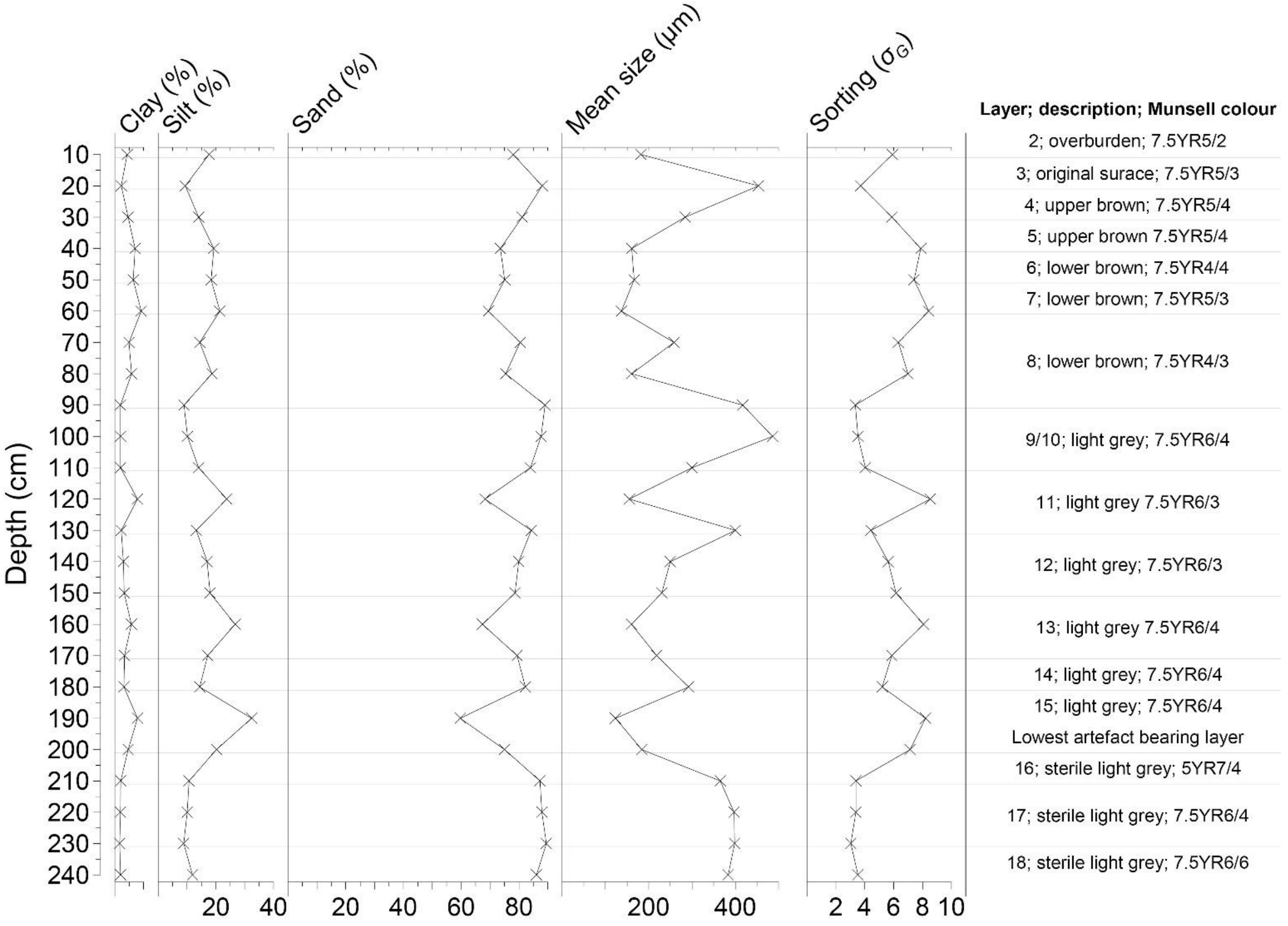

Supplementary Fig. 7

Relative abundances of sand/silt/clay sediment size fractions, mean grain size and degree of sorting, following geometric measures. Sand is the dominant size class throughout all samples. Sediments are all poorly sorted (2.00-4.00) or very poorly sorted (4.00-16.00).

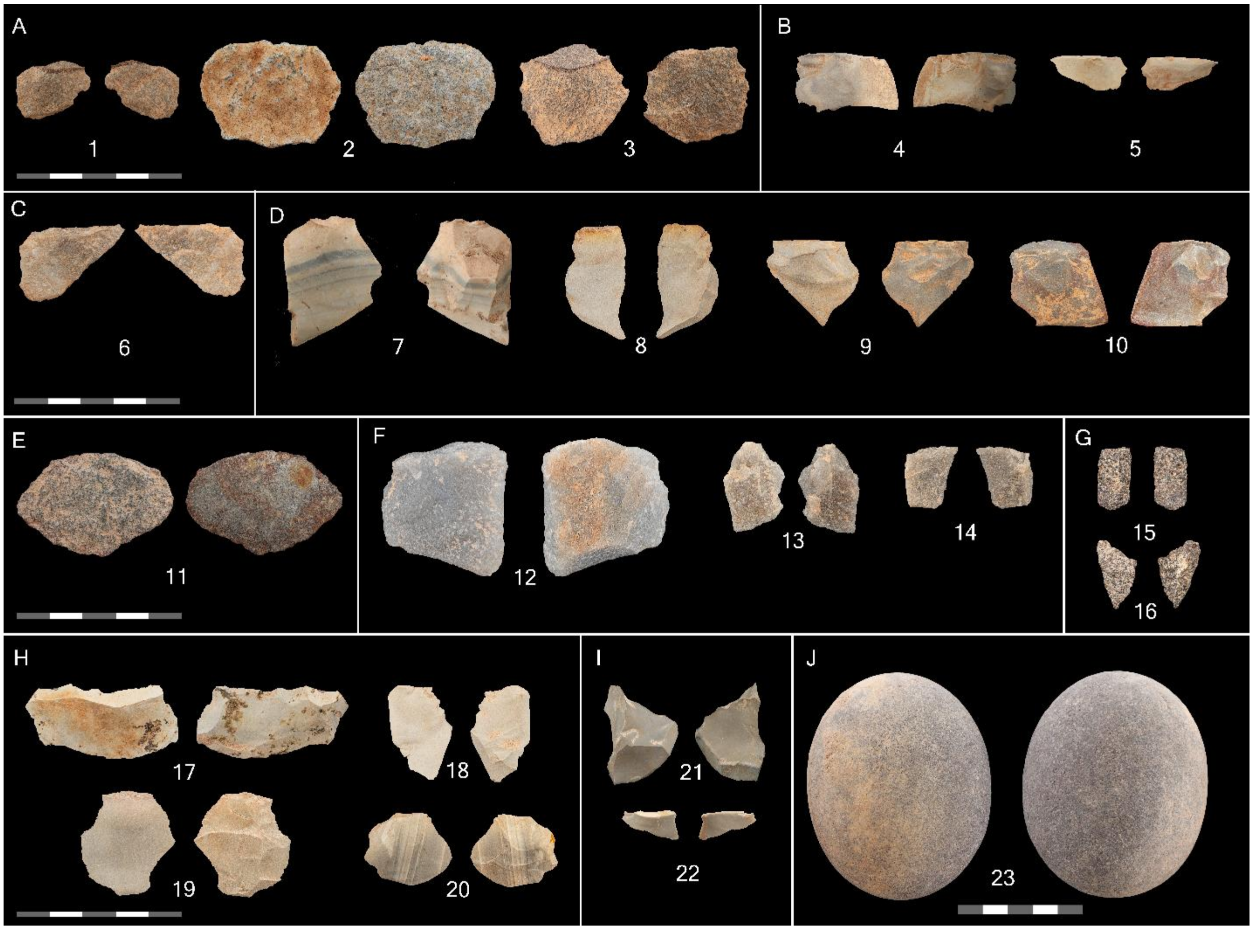

Supplementary Fig. 8

Stone artefacts excavated at Dargan Shelter, showing the range of different non-quartz raw materials present during the Holocene (A-D) and Late Pleistocene (E-J). (A)?quartzite; (B) exotic fine grained unidentified silicious stone possibly from Jenolan; (C) Holocene exotic course grained unidentified silicious stone possibly from Jenolan; (D) Local Burragorang claystone; (E) hornfels; (F) exotic course grained unidentified silicious stone possibly from Jenolan; (G) black quartzite; (H) Local Burragorang claystone; (I) exotic fine grained unidentified silicious stone possibly from Jenolan; (J) black quartzite hammerstone.

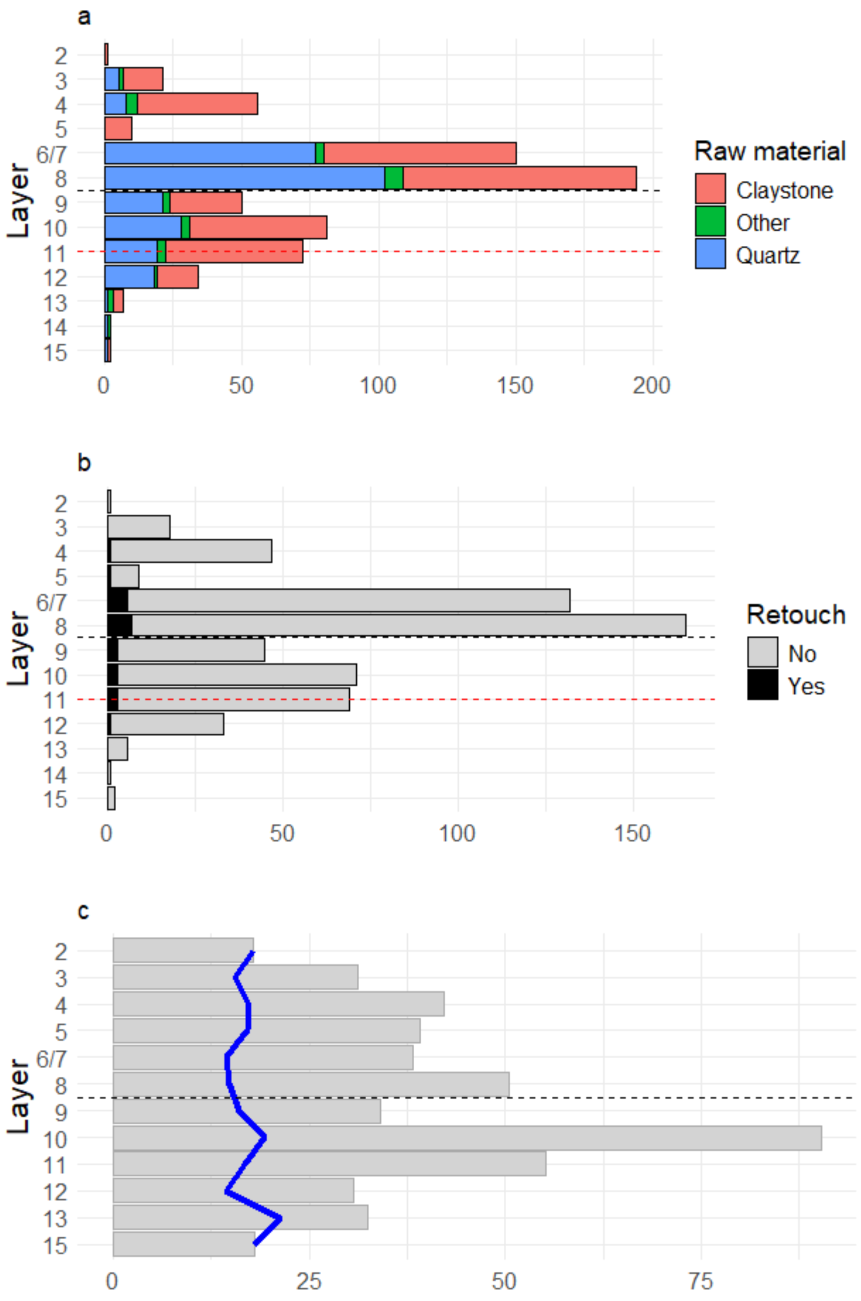

Supplementary Fig. 9

Stone artefact changes over time. The black dashed line marks the Holocene-Late Pleistocene transition, and the red dashed line the reduction in size of the excavation trench. (A) the raw material composition of each layer; (B) the number of retouched artefacts in each layer; (C) histogram showing maximum (bar plot) and average (line plot) length of complete flakes in each layer. For comparative artefact densities see Fig. 4.

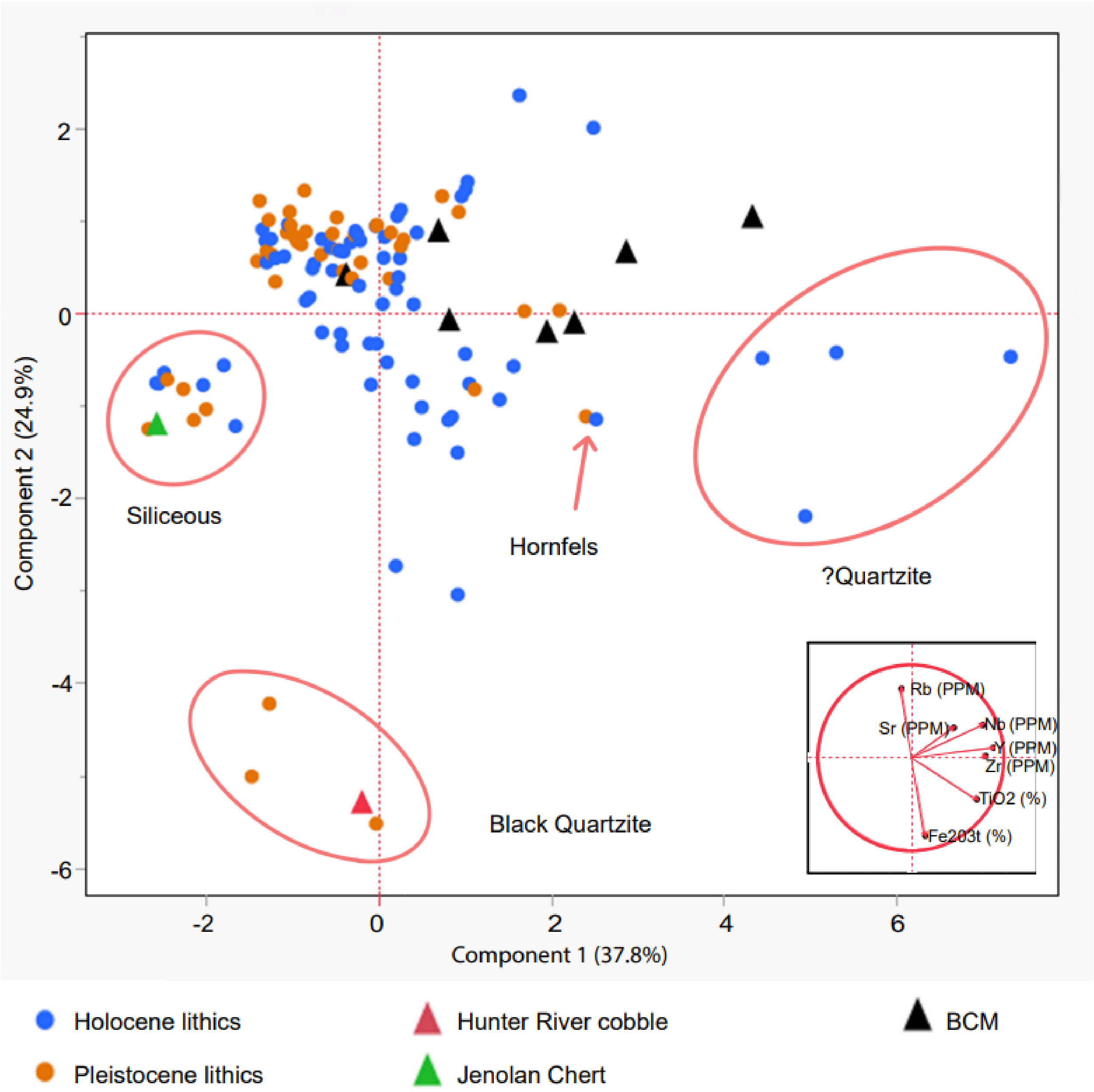

Supplementary Fig. 10

Multivariate (principal components) analysis showing the ferruginous sandstone, siliceous raw material groups, and the ?quartzite and hornfels lithics (circled). All other lithics are claystones, which have been compared to samples of the local BCM from the Australian Museum's geological collections. PCA variance is 62.7% (with 37.8 on the X axis and 24.9 on the Y axis).

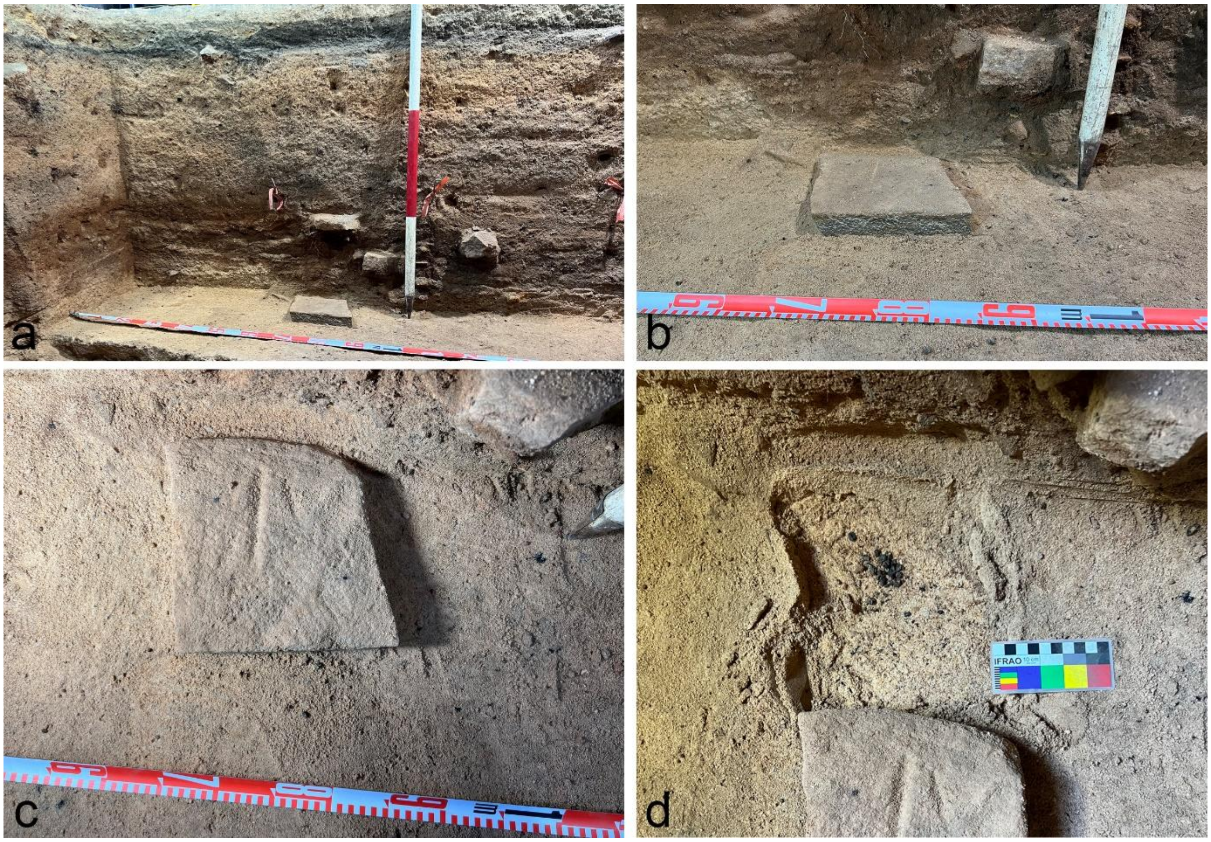

Supplementary Fig. 11

Sandstone slab during excavation. (A) showing position in section, (B) closeup of section showing sandstone slab positioned within light grey sand beneath the dark reddish brown Holocene unit, (C) close up *in situ* (D) showing underlying charcoal which dated to 13189-13460 cal. BP (S-ANU 74936).

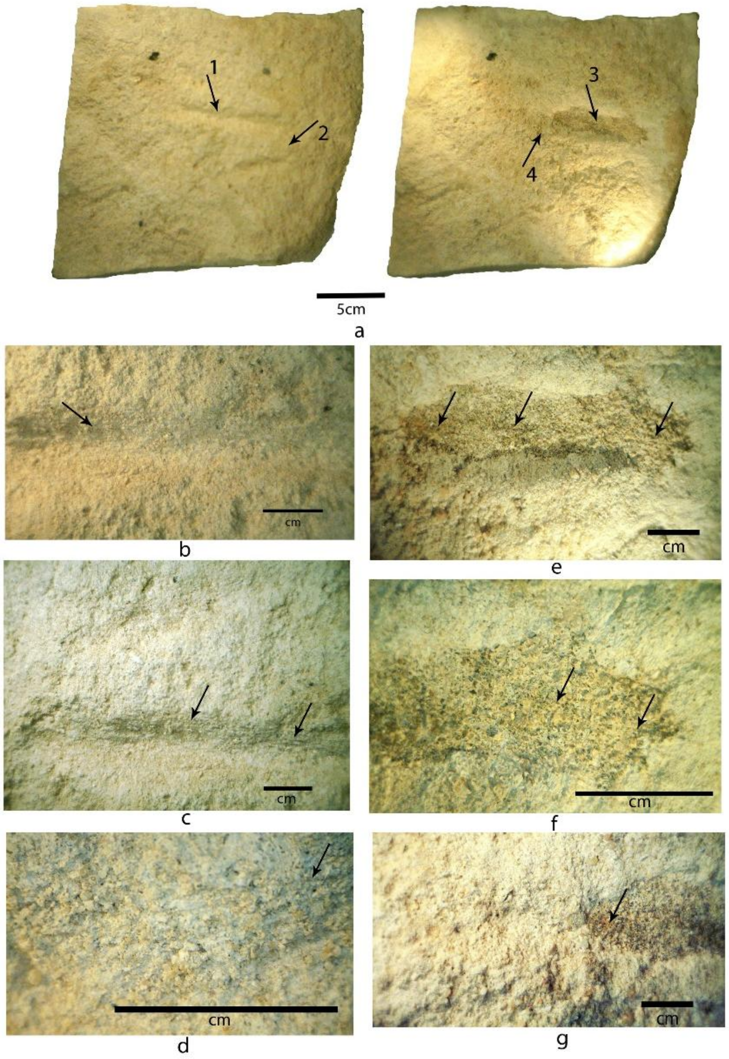

Supplementary Fig. 12

Use-wear results: sandstone block. (A) face of the plate before and after cleaning. Arrows indicates points 1-4 where images were taken; (B-D) the surface of the grooves before cleaning: (B) point 1, levelled smoothed surface with shallow parallel striations indicated by arrow, (C) point 2, short and long grooves with dense parallel striations indicated by arrows, (D) point 2, shallow parallel striations (×35); e-g – the surface of the grooves after cleaning: (E) point 3, surface of the groove after removing loose sandstone grains. The arrows indicate shallow parallel striations, (F) point 3, levelled rock grains and shallow parallel striations indicated by arrows (×20), (G) point 4, smoothed surface and shallow striations indicated by arrow.

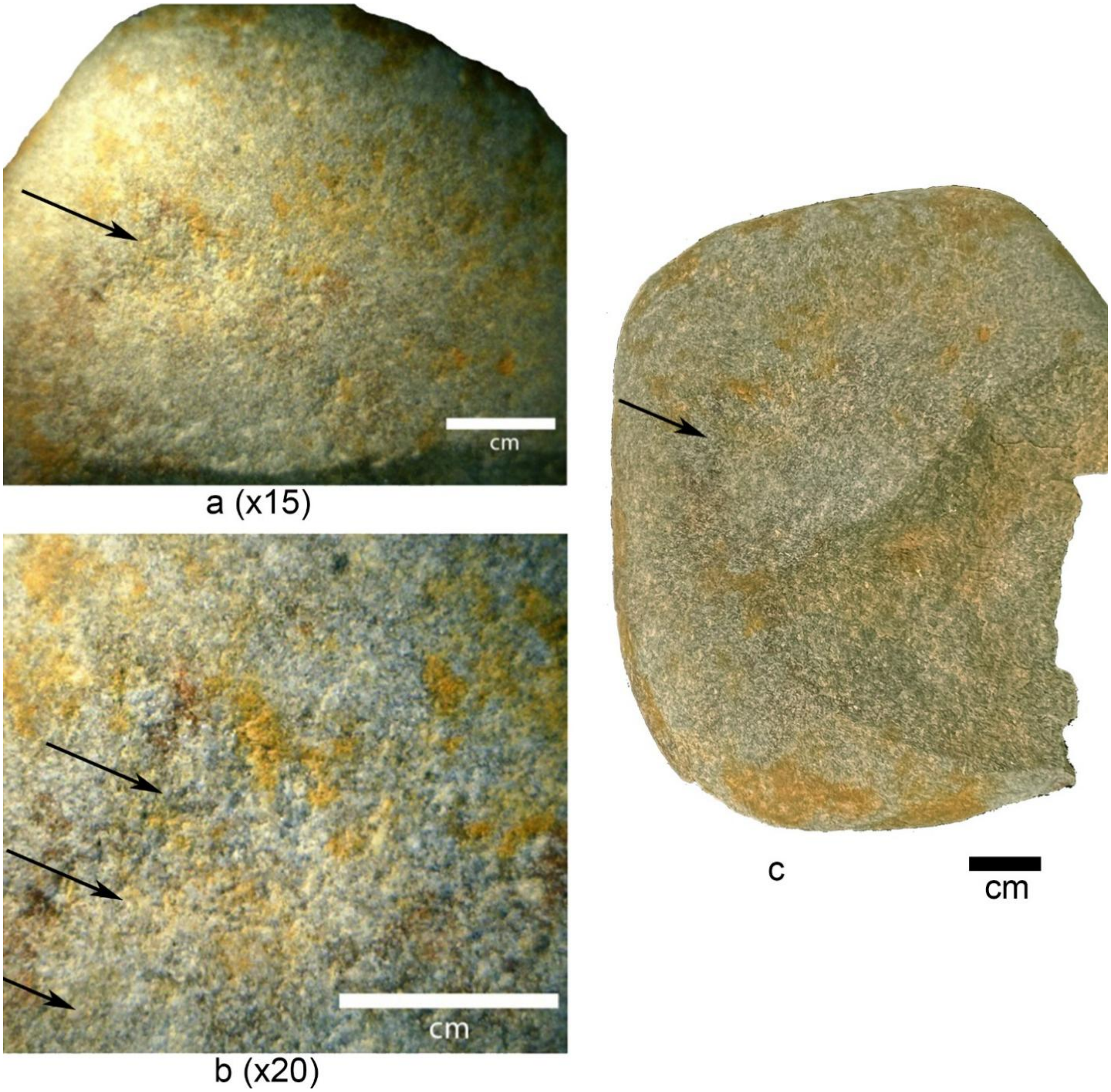

Supplementary Fig. 13

Use-wear results: basalt anvil. Wear on the surface: (A) surface lowering with dense impact marks (×15), (B) flattened rack grains, light sheen and fine striations indicated by arrows (×20). (C) location of use wear on anvil.

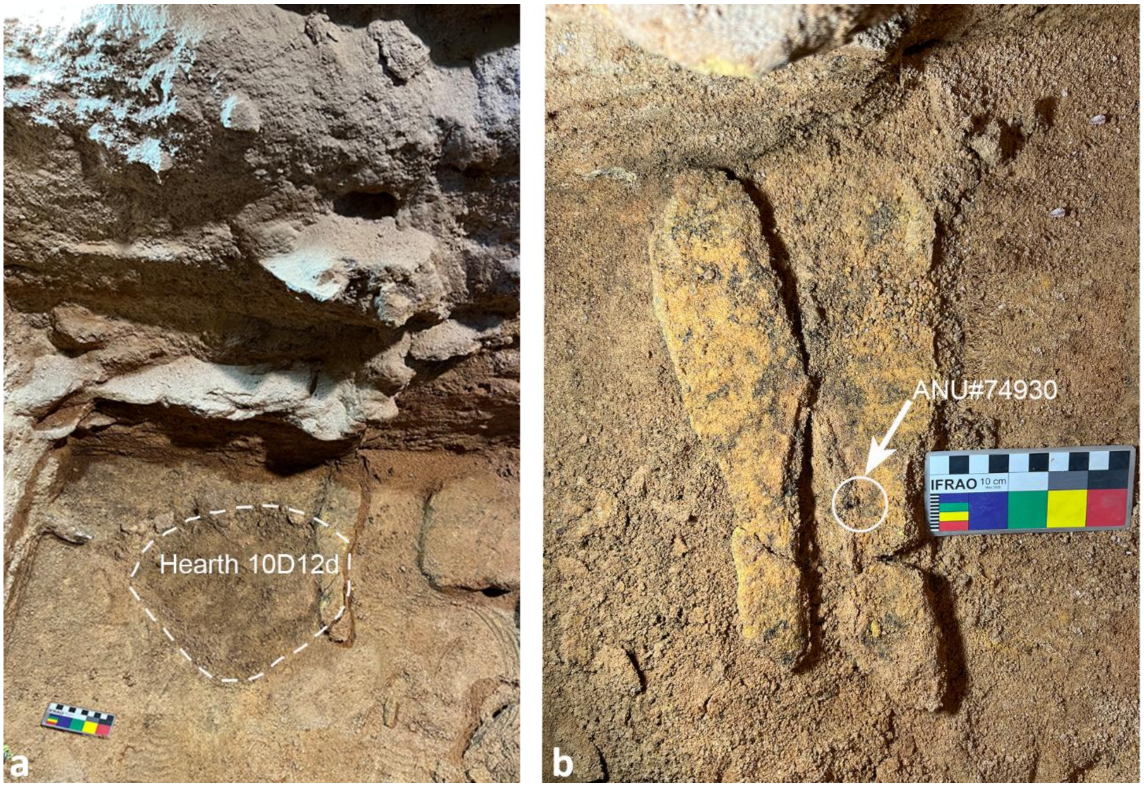

Supplementary Fig. 14

Lowest hearth at Dargan Shelter. (A) Photograph of Hearth 10D12d in pit 10D and at a depth below surface of 178cm showing stone overlying eastern margin. (B) photograph of carbon sample ANU#74930 located underneath marginal stone.

Supplementary Table 1

Previously excavated sites in Australia's Eastern Highlands showing site latitude (S), elevation (m), and ages. Note Birrigai reported a date of 25,290-25,070 cal. BP (Beta 16886), which was underlain by two radiocarbon dates of 18,300+1400/-1300 (ARL-162) and 13,400+4100/-2800 ^{14}C yr BP (ARL-145). The underlying ages could not be reliably calibrated due to the huge and asymmetric errors.

**Site name**	**Lat (S)**	**Elev (m)**	**C14 BP**	**Sample no.**	**Unmodelled (BP)**					**References**
					mu	Sig-ma	median	from_ 68_3	to_ 68_3	
Bone Cave	-42.8	400	29,000 ± 720	Beta-44081	33,263	850	33,294	34,200	32,255	[2–4]
Stone Cave	-42.8	400	18,110 ± 210	Beta-37557, ETH-6737	21,926	283	21,963	22,298	21,733	[2,3,5]
Nunamira Cave (formerly Bluff Cave)	-42.65	400	30,420 ± 690	Beta-25881	34,932	689	34,903	35,517	34,232	[2–4]
Tiata Mara Kominya a (Beginner's Luck Cave)	-42.6	400	20,650 ± 1790	GaK-7081	25,382	2308	25,140	27,043	22,965	[3,6]
ORS 7	-42.25	440	30,840 ± 480	Beta-23404, ETH-3724	35,238	468	35,207	35,609	34,650	[4]
Birrigai	-35.4	730	21,000 ± 220	Beta16 886	25,281	279	25,291	25,616	25,066	[7,8]
Birrigai	-35.4	730	13,400 +4100/-2800	ARL-145	*Could not be reliably calibrated due to the huge and asymmetric errors.*					
Birrigai	-35.4	730	18,300+ 1400/-1300	ARL-162	*Could not be reliably calibrated due to the huge error.*					
Kings Table	-33.74	872	14,534 ± 300	SUA-194	17,684	382	17,689	18,130	17,365	[9,10]
Lyrebird Dell	-33.71	985	12,550 ± 145	SUA-15	14,714	298	14,708	15,064	14,357	[10]
Wall's Cave	-33.65	910	12,000 ± 350	GaK-3448	14,077	511	13,992	14,790	13,447	[10]
Dargan Shelter	-33.5	1,073	16,816 ± 68	S-ANU 74930	20,290	100	20,296	20,405	20,203	this paper
Lidsdale, Coxs River	-33.35	810	13,500 ± 1000		16,313	1393	16,284	17,810	14,932	[11]
Warragarra	-41.8	610	10,600 ±450 *	Beta-8466	12,284	587	12,313	12,970	11,760	[12]

Little Thredbo 2	-36.5	1,200	4,390 ± 80	ANU-6867	4,987	141	4,961	5,210	4,836	[7]
Bunyan	-36.15	740	c. 7,000 years							[7]
Caddigat	-36	1,000	1,600 ± 60	ANU-1049	1,449	66	1,449	1,523	1,376	[7]
Yankee Hat 2	-35.7	1,098	>770 ± 140	ANU-1051	694	114	684	768	555	[7]
Yarrangobilly Y258	-35.65	1,100	8,668 ± 43	Wk-18839	9,607	57	9,597	9,660	9,537	[7]
Nursery Swamp 2	-35.6	1,200	3,700 ± 110	ANU-3033	4,007	162	4,003	4,150	3,840	[7]
Nursery Swamp 3	-35.6	1,215	970 ± 70	ANU-3953	836	67	838	918	772	[7]
Cooleman CP75	-35.55	1,200	1,330 ± 80	ANU-6191	1,186	83	1,197	1,290	1,096	[7]
Bogong 2	-35.5	1,433	1,000±60	ANU-1050	856	59	855	924	799	[7]
London Bridge BS1	-35.42	680	810 ± 170	ANU-7461	737	144	724	902	557	[7]
Hanging Rock	-35.4	823	370 ± 60	ANU-1047	390	65	393	455	320	[7]
Butmaroo 1	-35.2	700	>6,000 ± 700	ANU-3839	6,843	758	6,831	7,570	6,014	[7]
Sassafras 1	-35.15	730	3,770 ± 150	ANU-743	4,108	214	4,103	4,351	3,887	[7]
Sassafras 2	-35.15	730	>2,780 ± 115	ANU-744	2,883	148	2,874	2,997	2,747	[7]
Nardoo	-35.1	760	760 ± 110	ANU-1060	674	90	667	734	558	[7]
Bendemeer	-30.7	815	4,200							[13,14,15]
Church Gully	-30.6	1030	Late Holocene							[13,16]

Supplementary Table 2

The modelled ages (cal.yr BP) at 95% probability for Models A (General T-type) and B (Charcoal Plus) at Dargan Shelter. The dates are listed in stratigraphic order, with the oldest at the bottom.

Name	Model A (cal. BP)		C	Model B (cal. BP)		C
	from	to		from	to	
**End Dargan**	**500**	**-680**	**97.7**	**500**	**-770**	**99.5**
S-ANU72611	500	330	99.8	500	220	99.5
Start Layer 3	1400	340	98.5	1450	300	99.7
End Layer 4	1720	800	98.9	1720	790	99.6
S-ANU74931	1730	1570	99.5	1730	1470	99.6
S-ANU74938	1830	1620	99.6	1830	1530	99.4
Start Layer 4	2980	1620	98	2950	1540	99.3
End Layer 5	3880	2100	98.3	3870	2050	99.6
S-ANU71227	3900	3700	99.5	3920	3560	99.7
Start Layer 5	5000	3720	99.8	5040	3680	99.8
End Layer 6	5490	4430	98.9	5420	4450	99.6
S-ANU71229	5600	5330	99.6	5600	5210	99.1
S-ANU74937	5440	4980	99.4	5440	4960	99.8
S-ANU72610	5580	5330	99.4	5580	5190	99.2
Start Layer 6	5700	5480	99.8	5690	5280	99.3
End Layer 7	5750	5510	99.8	5750	5400	99.7
S-ANU71230	5840	5590	99.6	5850	5520	99.7
S-ANU72603	5900	5660	99.5	5910	5590	99.4
Start Layer 7	7030	5610	96.4	7080	5550	99
End Layer 8	8990	6510	97.2	8960	6500	99.4
S-ANU71231	10,120	9560	99.3	10,120	9500	99.5
S-ANU72609	8990	8640	99.3	8990	8570	99.7
S-ANU74935	9890	9540	99.4	9880	9420	99.5
Start Layer 8	12,120	9670	97.8	12,120	9580	99.5
End Layer 9	13,450	10,600	96.7	13,430	10,550	99.5
S-ANU74936	13,460	13,190	99.6	13,470	13,100	99.7
Start Layer 9	14,730	13,250	98.8	14,750	13,190	99.8
End Layer 10	15,190	13,740	98.8	15,170	13,720	99.8
S-ANU72604	15,250	14,900	99.5	15,250	14,790	99.7
Start Layer 10	16,030	14,970	98.8	16,030	14,910	99.8

End Layer 11	16,300	15,590	98.5	16,280	15,550	99.7
S-ANU72606	16,380	16,070	99.7	16,370	16,010	99.5
S-ANU72605	16,330	15,910	99.5	16,310	15,870	99.8
S-ANU72612	16,530	16,180	99.4	16,530	16,070	99.2
S-ANU72607	16,390	16,070	99.7	16,380	16,010	99.4
Start Layer 11	16,910	16,200	98.6	16,900	16,100	99.3
End Layer 12	17,760	16,560	98.4	17,740	16,510	99.6
S-ANU74928	17,830	17,280	99.5	17,810	17,190	99.7
S-ANU74929	17,950	17,410	99.4	17,970	17,340	99.7
Start Layer 12	18,560	17,460	98.7	18,550	17,410	99.7
End Layer 13	18,830	18,090	98.1	18,820	18,010	99.6
S-ANU74933	18,860	18,680	99.7	18,870	18,590	99.7
UNSW-12	18,840	18,710	99.8	18,850	18,610	99.7
Start Layer 13	19,620	18,720	98.7	19,650	18,640	99.6
End Layer 14	20,410	19,020	98.4	20,390	18,990	99.7
S-ANU74930	20,480	20,050	99.4	20,480	19,960	99.6
**Start Dargan**	**21,700**	**20,000**	**95.5**	**22,000**	**19,900**	**98.7**

Supplementary Table 3

The uncalibrated, unmodelled calibrated radiocarbon dates and F14C results with error margins recorded by ANU and UNSW sample number, pit and depth in the Dargan Shelter. All samples collected *in situ* during excavation.

Sample no. S-ANU#	Pit	Layer	Depth below surface (mm)	F14C	±	^{14}C age	Error	Unmodelled (cal. BP 95.4%) From To		Median_ cal. BP	Feature
72611	7E	3	152	0.9488	0.0020	422	22	500	330	462	Charcoal from hearth
74931	10D	4	242	0.8017	0.0025	1775	31	1710	1560	1647	Plotted charcoal
74938	13E	4	250	0.7926	0.0026	1868	31	1860	1630	1764	Charcoal from hearth
71227	5E	5	265	0.6420	0.0016	3560	25	3900	3700	3788	Plotted charcoal
74937	13D	6	370	0.5687	0.0025	4534	41	5310	4980	5155	Charcoal from hearth
72610	7E	6	438	0.5519	0.0015	4774	27	5580	5330	5486	Charcoal from hearth
71229	5E	6	355	0.5463	0.0015	4857	27	5600	5470	5525	Charcoal from hearth
71230	5E	7	450	0.5396	0.0014	4956	26	5720	5590	5641	Charcoal from hearth
72603	13D	7	450	0.5300	0.0020	5100	35	5910	5660	5814	Charcoal from hearth
72609	11D	8	640	0.3708	0.0012	7970	32	8990	8610	8776	Charcoal from hearth
74935	10F	8	732	0.3379	0.0017	8716	45	9890	9540	9633	Plotted charcoal

71231	5E	8	580	0.3332	0.0011	8828	32	10,120	9560	9810	Charcoal from hearth
74936	11C	9	770	0.2392	0.0014	11,492	53	13,460	13,190	13,340	Plotted charcoal
72604	13D	10	855	0.2042	0.0010	12,763	43	15,240	14,900	15,179	Plotted charcoal
72605	13E	11	1020	0.1894	0.0014	13,365	66	16,250	15,800	16,031	Charcoal from hearth
72606	13D	11	955	0.1862	0.0009	13,502	45	16,390	16,040	16,227	Charcoal from hearth
72607	12E	11	1073	0.1860	0.0009	13,511	46	16,410	16,050	16,240	Charcoal from hearth
72612	7D	11	1045	0.1834	0.0009	13,623	46	16,590	16,240	16,403	Plotted charcoal
74928	10D	12	1229	0.1674	0.0011	14,358	58	17,790	17,150	17,421	Plotted charcoal
74929	10D	12	1350	0.1628	0.0011	14,583	59	18,030	17,440	17,765	Plotted charcoal
74933	10E	13	1505	0.1454	0.0010	15,489	62	18,870	18,660	18,771	Plotted charcoal
UNSW-2345	7D	13	1600	0.1456	0.0005	15,500	30	18,850	18,700	18,782	Plotted charcoal
74930	10D	14	1780	0.1233	0.0010	16,816	68	20,480	20,090	20,296	Charcoal from hearth
74932	10D	18	2208	0.0020	0.0005	50,046	2153	Limits of radiocarbon		53,426	Plotted charcoal
ANU	10D	8	There is no usable material. Concreted ash with pebbles and small black fragments that are not charcoal.								
ANU	12D	16	There is no usable material. Concreted ash with pebbles and small black fragments that are not charcoal.								
ANU	11D	16	There is no usable material. Concreted ash with pebbles and small black fragments that are not charcoal.								
ANU	11D	16	In addition the above notes - One possible candidate was actually compressed sediment.								
UNSW	10D	15	There is no usable material. Concreted ash with pebbles and small black fragments that are not charcoal.								
UNSW	11D	15	There is no usable material. Concreted ash with pebbles and small black fragments that are not charcoal.								
UNSW	10E	16	There is no usable material. Concreted ash with pebbles and small black fragments that are not charcoal.								
UNSW	11E	18	There is no usable material. Concreted ash with pebbles and small black fragments that are not charcoal.								

Supplementary Table 4

Pollen results from Dargan Shelter.

Layer			Herbs			Trees/shrubs				Ferns				
	Depth (cm)	Lycopodium	Poaceae	Cyperaceae	Asteraceae	Podocarpus	Myrtaceae	Casaurina sp.	Ericaceae	Monolete	Trilete	Unid.	Total	Est. conc. cm^3
5	50	366		4					3			2	9	501
8	80	116	14	3	1	1	1	1	12	1	1	1	35	6157
11	110	220	5					1				1	7	649
12	150	279	7	1		1							9	658

Supplementary Table 5

Stone artefact counts by raw material and technological type from Dargan Shelter.

Layer	Claystone	Hornfels	Quartz	Quartzite	Sandstone	Siliceous	Unretouched Flake	Retouched Flake	Core	Flaked piece	Angular frag.	Hammer	Slab	Anvil	Total	Artefact Density/m2
**2**	1		0			0	1		0	0	0	0	0	0	1	0.13
**3**	14		5	1		1	18		0	0	3				21	2.80
**4**	44	1	8			3	46	1	1	1	7				56	7.47
**5**	10		0			0	8	1	0	0	1				10	1.33
**6+7**	70		77	1		2	126	6	5	4	9				150	20.00
**8**	85	1	102	2		4	158	7	3	6	19			1	194	25.87
**9**	26		21		1	2	42	3	0	1	3		1		50	6.67
**10**	50	1	28	1		1	68	3	4	0	5	1			81	12.00
**11**	50		19			3	66	3	1	1	1				72	13.71
**12**	15		18			1	32	1	1	0	0				34	11.33
**13**	4		1	2		0	6		1	0	0				7	2.33
**14**	0		1			1	1		1	0	0				2	0.67
**15**	1		1			0	2		0	0	0				2	0.67
**Total**	370	3	281	7	1	18	574	25	17	13	48	1	1	1	680	

Supplementary Table 6

pXRF raw material results showing counts by epoch.

Raw material and Epoch	Count of Raw Material
**Claystone**	90
Holocene	57
Late Pleistocene	33
**Ferruginous black quartzite**	3
Late Pleistocene	3
**Hornfels**	2
Holocene	1
Late Pleistocene	1
**Quartzite**	4
Late Pleistocene	1
Holocene	3
**Unidentified siliceous**	10
Holocene	5
Late Pleistocene	5
**Total**	109

Supplementary Table 7

pXRF calibration standards.

Standard	Description
SiO2 100%	Pure silica (international)
45d	Soil (international)
BHVO-1	Hawaiian basalt (international)
BHVO-2	Hawaiian basalt (international)
BIR-1	Icelandic basalt (international)
DNC-1 A	North Carolina diabase (international)
DNC-1 B	North Carolina diabase (international)
SBC-1	Brush Creek shale (international)
SCO-1	Cody shale (international)
SDC-1	Mica schist (international)
SGR-1	Green River shale (international)
STM-1	Nepheline syenite (international)
SARM-1 NIM-G	Granite (international)
JA-2	Japanese andesite (international)
JB-1b (1)	Japanese basalt (international)
JB-1b (2) / JB-1b (p)	Japanese basalt (international)
JB-2	Japanese basalt (international)
JB-3	Japanese basalt (international)
JG-3	Japanese granodiorite (international)
JR-1	Japanese rhyolite (international)
JR-2 (1)	Japanese rhyolite (international)
JR-2 (2) / JR-2 (p)	Japanese rhyolite (international)
JR-3	Japanese rhyolite (international)
JR-3 #3 / JR-3 (p)	Japanese rhyolite (international)
9.5 Obsidian	Obsidian
NBS688	Nevada basalt (international)
NIST688	Basalt (international)
NIST278	Obsidian (international)
NIST2710a	Soil (international)
NIST2711a	Soil (international)
ANU2000	Obsidian (in-house ANU)
GC-188	Tongan basalt (in-house ANU)
T-10	Tongan basalt (in-house ANU)
T-11	East Polynesian basalt (in-house ANU)
T-13	Tongan basalt (in-house ANU)
T-14	Samoan basalt (in-house ANU)

T-16	Fijian basalt (in-house ANU)
T-17	Samoan basalt (in-house ANU)
T-22	Samoan basalt (in-house ANU)
T-23	Tongan basalt (in-house ANU)
T-28	Samoan basalt (in-house ANU)
T-7	Samoan basalt (in-house ANU)
MQ001	Marqueasan basalt (in-house Auckland)
MQ007	Marqueasan phonolite (in-house Auckland)
MQ1529	Marqueasan basalt (in-house Auckland)
MQ5296	Marqueasan basalt (in-house Auckland)
MQ5337	Marqueasan basalt (in-house Auckland)
MQ5567	Marqueasan basalt (in-house Auckland)
MQ5875	Marqueasan basalt (in-house Auckland)

Supplementary code – age-models

Model A: General T-type Outlier Model Code

```
Plot()
 {
  Curve("SHCal20","shcal20.14c");
  Outlier_Model("General",T(5),U(0,4),"t");
  Sequence()
  {
   Boundary("Start XU15");
   Phase("XU15")
   {
    R_Date("S-ANU7930", 16816, 68)
    {
     Outlier(0.05);
    };
   };
   Boundary("End XU15");
   Boundary("Start XU13");
   Phase("XU13")
   {
    R_Date("UNSW-12", 15500, 30)
    {
     Outlier(0.05);
    };
    R_Date("S-ANU74933", 15489, 62)
    {
     Outlier(0.05);
    };
   };
   Boundary("End XU13");
   Boundary("Start XU12");
   Phase("XU12")
   {
    R_Date("S-ANU74929",14583,59)
    {
     Outlier(0.05);
    };
    R_Date("S-ANU74928",14358,58)
    {
     Outlier(0.05);
    };
   };
   Boundary("End XU12");
   Boundary("Start XU11");
```

```
Phase("XU11")
{
 R_Date("S-ANU72607",13511,46)
 {
  Outlier(0.05);
 };
 R_Date("S-ANU72612",13623,46)
 {
  Outlier(0.05);
 };
 R_Date("S-ANU72605",13365,66)
 {
  Outlier(0.05);
 };
 R_Date("S-ANU72606",13502,45)
 {
  Outlier(0.05);
 };
};
Boundary("End XU11");
Boundary("Start XU10");
Phase("XU10")
{
 R_Date("S-ANU72604",12673,43)
 {
  Outlier(0.05);
 };
};
Boundary("End XU10");
Boundary("Start XU9");
Phase("XU9")
{
 R_Date("S-ANU74936",11492,53)
 {
  Outlier(0.05);
 };
};
Boundary("End XU9");
Boundary("Start XU8");
Phase("XU8")
{
 R_Date("S-ANU74935",8716,45)
 {
  Outlier(0.05);
 };
 R_Date("S-ANU72609",7970,32)
```

```
 {
  Outlier(0.05);
 };
 R_Date("S-ANU71231",8828,32)
 {
  Outlier(0.05);
 };
};
Boundary("End XU8");
Boundary("Start XU7");
Phase("XU7")
{
 R_Date("S-ANU72603",5100,35)
 {
  Outlier(0.05);
 };
 R_Date("S-ANU71230",4956,26)
 {
  Outlier(0.05);
 };
};
Boundary("End XU7");
Boundary("Start XU6");
Phase("XU6")
{
 R_Date("S-ANU72610",4774,27)
 {
  Outlier(0.05);
 };
 R_Date("S-ANU74937",4534,41)
 {
  Outlier(0.05);
 };
 R_Date("S-ANU71229",4857,27)
 {
  Outlier(0.05);
 };
};
Boundary("End XU6");
Boundary("Start XU5");
Phase("XU5")
{
 R_Date("S-ANU71227",3560,25)
 {
  Outlier(0.05);
 };
```

```
  };
  Boundary("End XU5");
  Boundary("Start XU4");
  Phase("XU4")
  {
   R_Date("S-ANU74938",1868,31)
   {
    Outlier(0.05);
   };
   R_Date("S-ANU74931",1775,31)
   {
    Outlier(0.05);
   };
  };
  Boundary("End XU4");
  Boundary("Start XU3");
  Phase("XU3")
  {
   R_Date("S-ANU72611",422,22)
   {
    Outlier(0.05);
   };
  };
  Boundary("End XU3");
 };
};
```

Model B: Modified Charcoal or Charcoal Plus Model Code

```
Plot()
 {
  Curve("SHCal20","shcal20.14c");
  Outlier_Model("Charcoal_Plus",Prior("charcoal_plus"),U(0,3),"t");
  Sequence()
  {
   Boundary("Start XU15");
   Phase("XU15")
   {
    R_Date("S-ANU7930", 16816, 68)
    {
     Outlier(1);
    };
   };
   Boundary("End XU15");
```

```
Boundary("Start XU13");
Phase("XU13")
{
 R_Date("UNSW-12", 15500, 30)
 {
  Outlier(1);
 };
 R_Date("S-ANU74933", 15489, 62)
 {
  Outlier(1);
 };
};
Boundary("End XU13");
Boundary("Start XU12");
Phase("XU12")
{
 R_Date("S-ANU74929",14583,59)
 {
  Outlier(1);
 };
 R_Date("S-ANU74928",14358,58)
 {
  Outlier(1);
 };
};
Boundary("End XU12");
Boundary("Start XU11");
Phase("XU11")
{
 R_Date("S-ANU72607",13511,46)
 {
  Outlier(1);
 };
 R_Date("S-ANU72612",13623,46)
 {
  Outlier(1);
 };
 R_Date("S-ANU72605",13365,66)
 {
  Outlier(1);
 };
 R_Date("S-ANU72606",13502,45)
 {
  Outlier(1);
 };
};
```

```
Boundary("End XU11");
Boundary("Start XU10");
Phase("XU10")
{
 R_Date("S-ANU72604",12673,43)
 {
  Outlier(1);
 };
};
Boundary("End XU10");
Boundary("Start XU9");
Phase("XU9")
{
 R_Date("S-ANU74936",11492,53)
 {
  Outlier(1);
 };
};
Boundary("End XU9");
Boundary("Start XU8");
Phase("XU8")
{
 R_Date("S-ANU74935",8716,45)
 {
  Outlier(1);
 };
 R_Date("S-ANU72609",7970,32)
 {
  Outlier(1);
 };
 R_Date("S-ANU71231",8828,32)
 {
  Outlier(1);
 };
};
Boundary("End XU8");
Boundary("Start XU7");
Phase("XU7")
{
 R_Date("S-ANU72603",5100,35)
 {
  Outlier(1);
 };
 R_Date("S-ANU71230",4956,26)
 {
  Outlier(1);
```

```
 };
};
Boundary("End XU7");
Boundary("Start XU6");
Phase("XU6")
{
 R_Date("S-ANU72610",4774,27)
 {
  Outlier(1);
 };
 R_Date("S-ANU74937",4534,41)
 {
  Outlier(1);
 };
 R_Date("S-ANU71229",4857,27)
 {
  Outlier(1);
 };
};
Boundary("End XU6");
Boundary("Start XU5");
Phase("XU5")
{
 R_Date("S-ANU71227",3560,25)
 {
  Outlier(1);
 };
};
Boundary("End XU5");
Boundary("Start XU4");
Phase("XU4")
{
 R_Date("S-ANU74938",1868,31)
 {
  Outlier(1);
 };
 R_Date("S-ANU74931",1775,31)
 {
  Outlier(1);
 };
};
Boundary("End XU4");
Boundary("Start XU3");
Phase("XU3")
{
 R_Date("S-ANU72611",422,22)
```

```
   {
    Outlier(1);
   };
  };
  Boundary("End XU3");
 };
};
```

Supplementary References

1. Wellman, P. Eastern Highlands of Australia; their uplift and erosion. *Geosci. Aust. Rec.* **10**, 277–286 (1987).
2. Allen, J. Report of the Southern Forests Archaeological Project, vol. 1: Site Descriptions, Stratigraphies and Chronologies. *Melb. Sch. Archaeol. Trobe Univ.* (1996).
3. Gilligan, I. Resisting the cold in ice age Tasmania: thermal environment and settlement strategies. *Antiquity* **81**, 555–568 (2007).
4. Cosgrove, R. Forty-Two Degrees South: The Archaeology of Late Pleistocene Tasmania. *J. World Prehistory* **13**, 357–402 (1999).
5. Allen, J. & Cosgrove, R. Stone Cave. in *Report of the Southern Forests Archaeological Project* vol. 1 123–33 (La Trobe University, Bundora, 1996).
6. Murray, P. F., Goede, A. & Bada, J. L. Pleistocene Human Occupation at Beginners Luck Cave, Florentine Valley, Tasmania. *Archaeol. Phys. Anthropol. Ocean.* **15**, 142–152 (1980).
7. Theden-Ringl, F. Aboriginal presence in the high country: new dates from the Namadgi Ranges in the Australian Capital Territory. *Aust. Archaeol.* **82**, 25–42 (2016).
8. Flood, J., David, B., Magee, J. & English, B. Birrigai: a Pleistocene site in the south-eastern highlands. *Archaeol. Ocean.* **22**, 9–26 (1987).

9. Stockton, E. & Holland, W. Cultural sites and their environment in the Blue Mountains. *Archaeol. Phys. Anthropol. Ocean.* **9**, 36–65 (1974).
10. Stockton, E. New Discoveries. in *Blue Mountains Dreaming: The Aboriginal Heritage* (eds. Stockton, E. & Merriman, J.) (Blue Mountain Education and Research Trust, Lawson, 2009).
11. Benton, J. & Cameron, P. *Indigenous Heritage Assessment for Proposed 1.7km, 11kV Powerline Corridor and Ventilation Fan Compound, Baal Bone Colliery - Ben Bullen State Forest, Report Prepared by OzArk Environmental and Heritage Management Pty Ltd for Umwelt Australia*. (2007).
12. Allen, J. & Porch, N. Warragarra Rockshelter. in *Report of the Southern Forests Archaeological Project, vol. 1: Site Descriptions, Stratigraphies and Chronologies* 195–218 (La Trobe University, Bundora, 1996).
13. Godwin, L. Inside Information: Settlement and Alliance in the Late Holocene of North-eastern NSW. (University of New England, Armidale, 1990).
14. McBryde, I. Determinants of assemblage variation in New England prehistory. in *Stone tools as cultural markers* (ed. Wright, R. V. S.) vol. 12 223–225 (Australian Institute of Aboriginal and Torres Strait Islander Studies (AIATSIS), Canberra, 1977).
15. McBryde, I. Radiocarbon dates for northern New South Wales. *Antiquity* **40**, 285–292 (1966).
16. Bowdler, S. Hunters in the Highlands: Aboriginal Adaptations in the Eastern Australian Uplands. *Archaeol. Ocean.* **16**, 99–111 (1981).
17. Martin, L., Goff, J., Jacobsen, G. & Mooney, S. The Radiocarbon Ages of Different Organic Components in the Mires of Eastern Australia. *Radiocarbon* **61**, 173–184 (2019).

18. Robbie, A. & Martin, H. A. The history of the vegetation from the last glacial maximum at Mountain Lagoon, Blue Mountains, New South Wales. in *Proceedings of the Linnean Society of New South Wales* vol. 128 57–80 (2007).

19. Chalson, J. M. & Martin, H. A. A Holocene History of the Vegetation of the Blue Mountains, New South Wales. *Proc. Linn. Soc. New South Wales* **130**, 77–109 (2009).

20. Schneider, M. A. *et al.* Long-term mercury accumulation and climate reconstruction of an Australian alpine lake during the late Quaternary. *Glob. Planet. Change* **240**, 104539 (2024).

21. Kemp, J. & Hope, G. Vegetation and environments since the Last Glacial Maximum in the Southern Tablelands, New South Wales. *J. Quat. Sci.* **29**, 778–788 (2014).

47. Kohen, J. L., Stockton, E. D. & Williams, M. A. Shaws Creek KII rockshelter: a prehistoric occupation site in the Blue Mountains piedmont, eastern New South Wales. *Archaeol. Ocean.* **19**, 57–73 (1984).
48. McLaren, A. & Oakes, G. Late Holocene technological provisioning at the Kings Table rockshelter, Blue Mountains, New South Wales, Australia. *Archaeol. Ocean.* **58**, 227–244 (2023).
49. Stockton, E. & Holland, W. Cultural sites and their environment in the Blue Mountains. *Archaeol. Phys. Anthropol. Ocean.* **9**, 36–65 (1974).
50. Bamberry, W. J. Stratigraphy and sedimentology of the late Permian Illawarra coal measures, Southern Sydney Basin, New South Wales. (The University of Wollongong, Wollongong, 1991).
51. Branagan, D. F., Pickett, J. & Percival, I. G. Geology and geomorphology of Jenolan Caves and the surrounding region. *Proc. Linn. Soc. New South Wales* **136**, 99–130 (2014).
52. Grave, P., Attenbrow, V., Sutherland, L., Pogson, R. & Forster, N. Non-destructive pXRF of mafic stone tools. *J. Archaeol. Sci.* **39**, 1674–1686 (2012).
53. Hatherly, P. J. Landscape evolution of the Blue Mountains revealed by longitudinal river profiles and Cenozoic basalts and gravels. *Aust. J. Earth Sci.* **67**, 243–263 (2020).
54. Kononenko, N., Attenbrow, V., White, P., Asmussen, B. & Torrence, R. Cracking seeds and nuts: Replicating use-wear on pitted ground-edged stone hatchets from Southeastern Australia. *J. Archaeol. Sci. Rep.* **37**, 102994 (2021).
55. Attenbrow, V. & Kononenko, N. Microscopic revelations: The forms and multiple uses of ground-edged artefacts of the New South Wales Central Coast. *Tech. Rep. Aust. Mus. Online* **29**, 1–100 (2019).

56. Hayes, E., Pardoe, C. & Fullagar, R. Sandstone grinding/pounding tools: Use-trace reference libraries and Australian archaeological applications. *J. Archaeol. Sci. Rep.* **20**, 97–114 (2018).

57. Attenbrow, V. *Sydney's Aboriginal Past: Investigating the Archaeological and Historical Records*. (UNSW Press, 2010).

58. Taçon, P. S., Brennan, W., Hooper, S., Kelleher, M. & Pross, D. Greater Wollemi: a new Australian rock-art area bordering Sydney. *INORA Int. Newsl. Rock Art* **4**, p-01 (2005).
